# A rare case report of infant ileal atresia with double appendix

**DOI:** 10.1016/j.ijscr.2020.08.023

**Published:** 2020-08-29

**Authors:** Pinak Pani Dhar, Upasana Mohanty

**Affiliations:** Department of General Surgery, Silchar Medical College & Hospital, Silchar, India

**Keywords:** Ileal atresia, Double appendix, Male infants, Case report

## Abstract

**Introduction:**

Small bowel atresia has quite an incidence in infants particularly in males. It is usually seen to be the most common cause of bowel obstruction in neonates since birth. It may be associated with other congenital anomalies.

**Presentation of the case:**

A 4 day old neonate presented with abdominal distension, billous vomiting & non passage of stools since birth. On further examination, it was seen that the abdominal distension was mostly central & there was hyperperistaltic bowel sounds on auscultation. The infant also had scoliosis. There was decreased appetite & features of dehydration. Diagnosed by USG(W/A), infantogram & X-Ray plain picture abdomen.

**Discussion:**

The mortality from the atresia of ileum is greater owing to the incidence of early perforation. Infants may present with features discussed above. They usually present during the 1st week of life but majority of the babies presenting late have significant mortality.

Early surgical intervention with excision of the dilated bowel segment with anastomosis of the proximal (normal caliber bowel) to the distal part of the bowel has been seen to have been associated with decreased morbidity & mortality.

**Conclusion:**

It has been observed that one of the many causes for neonatal intestinal obstruction is attributed to small bowel atresia. This has been associated with successful outcome depending on the presentation of the child to the hospital to the timing of surgical intervention along with resuscitation & starting of enteral feeds. Small bowel atresia can be associated with antenatal morbidity in the mother as cited here.

## Highlights

• Study deals with rare case of ileal atresia with double appendix & associated congenital anomalies.
• Chromosomal & genetic typing of the infant is necessary as he had other associated anomalies.
• Study dealt with resection & anastomosis of the bowel & the surgical outcome depends on the presentation of the baby & the time taken to initiate surgical intervention.
• Post op care & regular follow up determined the success of the surgical intervention.

## 1 Introduction

Terminal ileal atresia occurs in 1 in 3000 live [[1], [2], [3], [4], [5], [6], [7], [8], [9], [10]] births of infant population [1]. It is associated with billous vomiting, abdominal distension & non passage of meconium since birth. It is associated with underlying dehydration & electrolyte abnormalities.

This is a case report of congenital terminal ileal atresia with double appendix in a male infant. The work has been reported in line with the SCARE criteria [9].

## 2 Case report

A 4 day old male infant presented with abdominal distension, billous vomiting & non passage of meconium since birth to our hospital. The parents also reported that the infant would have frequent episodes of billous vomiting immediately following breast feeding.

**Fig. 1:**
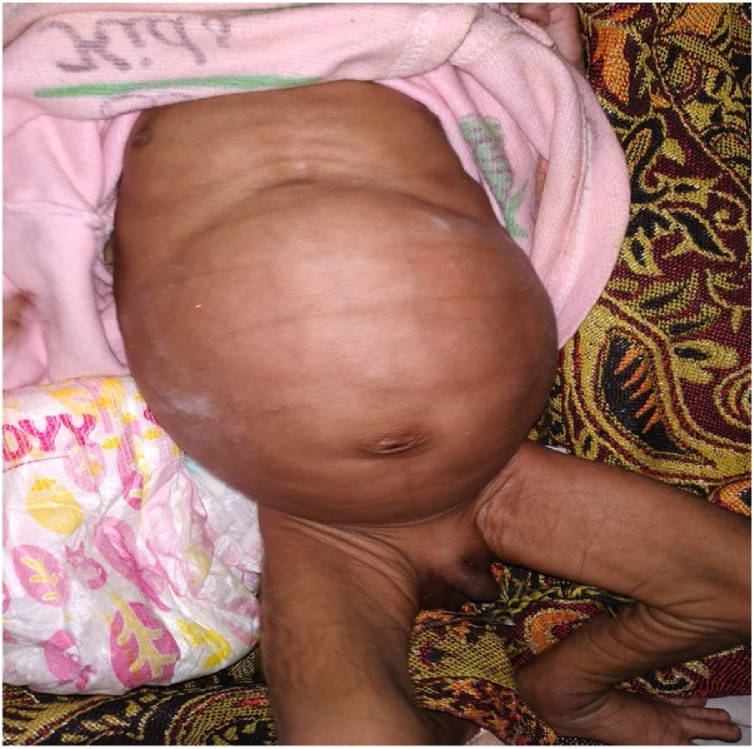
Inspection of abdomen.

On examination, the abdomen was distended & there was hyperperistaltic bowel sounds (Fig. 1). The anal opening was present & on per rectal examination, uniform ballooning of rectum was felt. On further enquiry it was learned that it was a case of pre-term normal vaginal delivery associated with antenatal gestational diabetes mellitus.

## 3 Methods

After thorough clinical evaluation, the infant was made to undergo several radiological investigations like USG (whole abdomen) & an infantogram. The patient’s perspective included a thorough holistic care in terms of surgical care, genetic & chromosomal typing,regular follow up to treatment adherence & tolerability.

USG of whole abdomen revealed dilated large bowel loops with collapsed small bowel associated with decreased peristalsis & normal vascularity suggestive of intestinal obstruction.

**Fig. 2:**
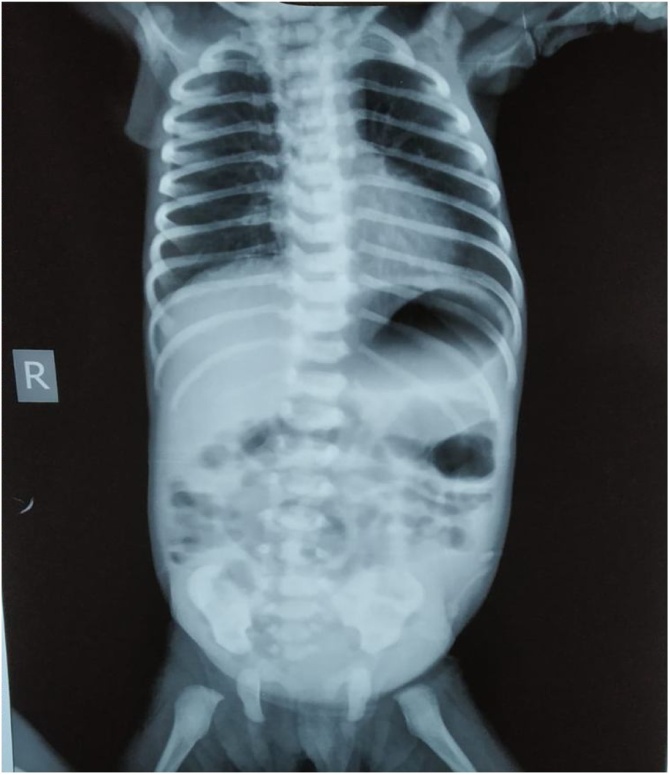
X-ray plain picture abdomen of the infant.

X- ray plain picture abdomen revealed dilated bowel loops in the centre with absence of gas shadow in the pelvis (Fig. 2). In blood picture, the total counts were elevated & serum sodium & potassium were found to be decreased.

The patient’s mother was a case of gestational diabtetes mellitus & was put on regular insulin. There was no significant family or genetic history. The mother was an elderly primigravida.

General examination of the baby revealed severe dehydration, scoliosis & right ectopic kidney.

After a thorough clinical, pathological & radiological intervention the infant was planned to be taken up for emergency OT. The procedure was performed under GA. Surgery was lead by the principal author(1st author)of this case report who is an Assistant Professor in the department of general surgery & the corresponding author was the assistant.

**Fig. 3:**
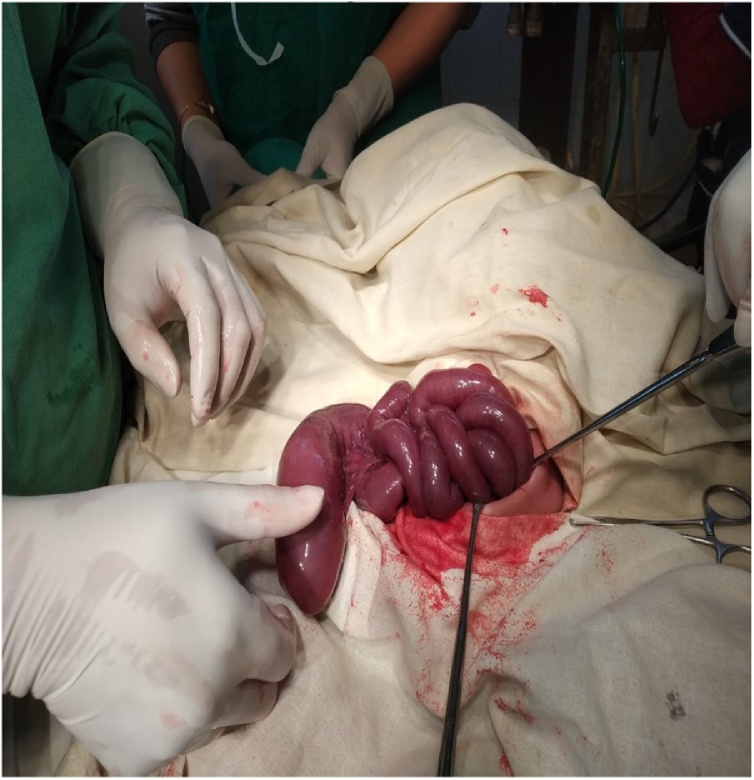
Exploratory lapatomy showing blind ending terminal ileum.

**Fig. 4:**
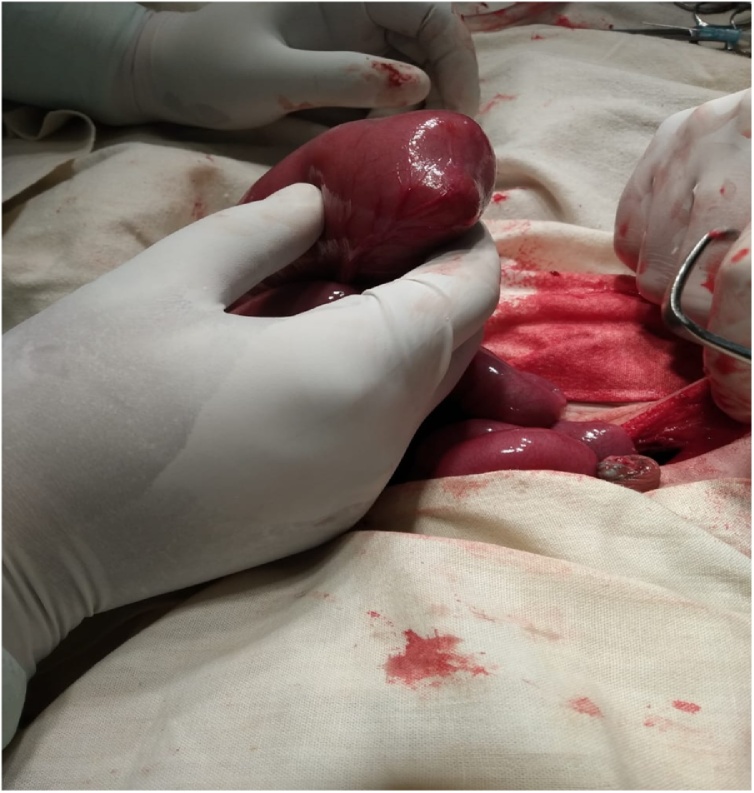
Blind ending terminal ileum.

**Fig. 5:**
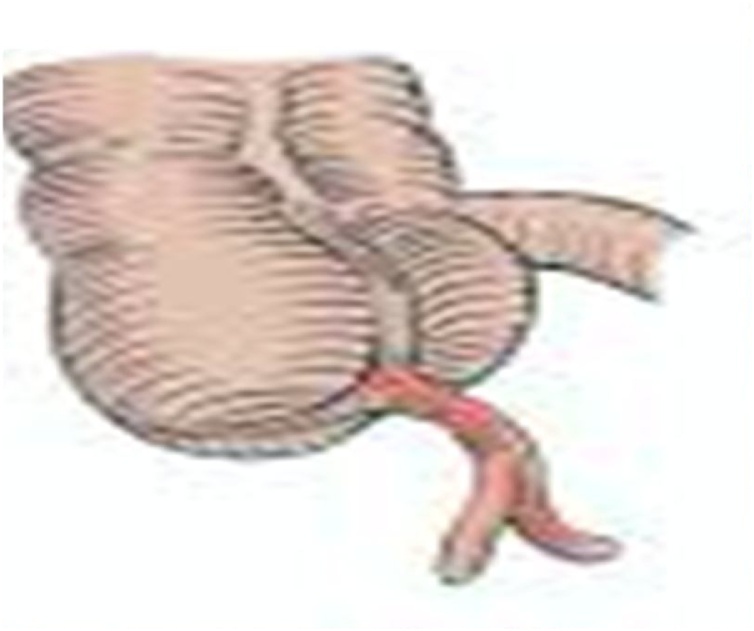
Bird beak appendix.

**Fig. 6:**
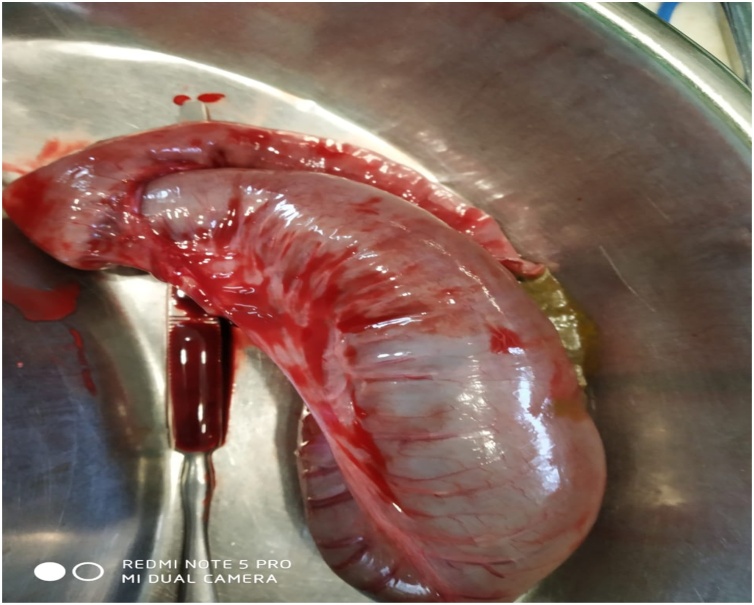
Resected segment of terminal ileum.

**Fig. 7:**
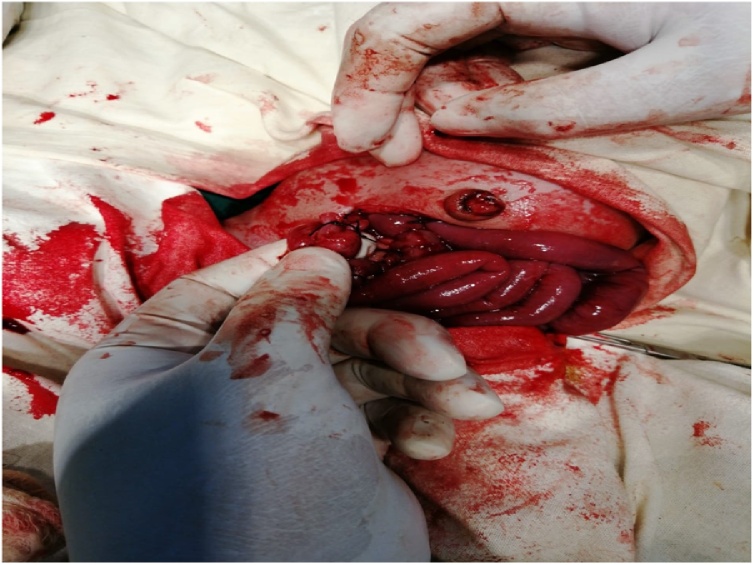
Resection & anastomosis of proximal with distal ileum.

Surgery revealed small bowel atresia with dilated, blind ending terminal ileum (Figs. 3 & 4). The base of the caecum revealed a bird beak (double) appendix (Fig. 5). Terminal ileum was resected & proximal Ileo-cecal anastomosis with double appendicectomy was done (Figs. 6 & 7).

The work has been reported in line with SCARE 2018 criteria [9]. The study has been registered at Research Registry bearing an UIN no- researchregistry5588 [10].

## 4 Discussion

Intestinal atresias are the most common cause of intestinal obstruction in infants comprisning of approximately 22.4% [2]. The mortality from the atresia of ileum is greater than other atresias in any part of bowel owing to the incidence of early perforation [3]. Important differential diagnosis includes Hirschsprung disease, malrotation of gut, duplication cyst, duodenal atresia, meconium ileus, obstructed hernia etc. Gestational age & birth weight play an important role in neonatal surgical outcome [4]. Among all the cases of atresia, jejunoileal atresia predominate [5]. Atresia is diagnosed by USG(W/A), infantogram & X-Ray plain picture abdomen.

Infants may present with abdominal distension, billous vomiting, inability to pass meconium with severe dehydration & electrolyte imbalance. They usually present during the 1st week of life but majority of the babies presenting late have significant mortality [6].

The mortality associated with neonatal intestinal obstruction ranges between 21% and 45% in developing countries, unlike less than 15% in Europe [7].

Early surgical intervention with excision of the dilated segment of the bowel with anastomosis helps in salvaging the complications associated with it. Associated conditions like sepsis, hypothermia, nutrition & wound care needs to be taken care of [8]. Early exploration of abdomen with concomitant correction of sepsis, nutrition, hypothermia & post-op vitals & input/output monitoring is to initiated so as to expect a better outcome.

In this case the infant was discharged on 12th post operative day after initiation of oral feeds & passage of stools. Considering the history of GDM in the mother & the associated congenital anomalies in the infant, he was subjected to chromosomal & genetic typing at a higher centre due to limitation of facilities at our setup; post operatively. The parents were advised for monthly follow up for six months. The infant had tolerated the surgery well & was thriving well.

## 5 Conclusion

It has been observed that one of the many causes for neonatal intestinal obstruction is attributed to small bowel atresia. These cases of small bowel atresia may have associated congenital anomalies, more so if there is an underlying antenatal morbidity in the mother. Hence a holistic care in terms of genetic & chromosomal typing along with surgery is necessary. Early surgical management of the case with resection anastomosis with early initiation of enteral feeding has been associated with successful outcome. The degree of success depends on the presentation of the infant to the hospital to the timing of surgical intervention. Involvement of other superspecialities has a cornerstone in the development & the determining the outcome of surgical procedure insuch infants having other associated congenital anomalies.

## Declaration of Competing Interest

No conflict of interest including employment, consultancies, stock ownership, patent applications, grants or other fundings.

## Funding

No funds for the research. Since it was a case report (single participant) I bore the expenses.

## Ethical approval

Since the study was carried out at the time of covid pandemic in India in the month of April 2020 & as the infant had to undergo emergency exploratory laparotomy with resection anastomosis of the bowel, the members at the institutional ethical committee had no meeting in view of the pandemic & had to told to go on with the study since there was no objection raised when mailed to them. Hence no ref no was attached.

## Consent

Written informed consent was obtained from the patient & his parents for the surgical procedure & for the publication of this case report along with the accompanying images. A copy of written consent is available for review by Editor-in-Chief of this journal on request.

## Author contribution

Pinak Pani Dhar- Conceptualisation, operative procedure, supervision, editing, visualisation, validation.

Upasana Mohanty- Conceptualisation, methodology, data entry, writing the manuscript, editing, data analysis & interpretation.

## Registration of research studies

1. Name of the registry: Research Registry.
2. Unique identifying number or registration ID: researchregistry5588.
3. Hyperlink to your specific registration (must be publicly accessible and will be checked): https://www.researchregistry.com/browse-the-registry#home/.

## Guarantor

Dr Upasana Mohanty.

## Provenance and peer review

Not commissioned, externally peer-reviewed.
